# Investigating the impact of primary care networks on continuity of care in English general practice: Analysis of interviews with patients and clinicians from a mixed methods study

**DOI:** 10.1111/hex.14032

**Published:** 2024-03-31

**Authors:** Mhorag Goff, Sally Jacobs, Jonathan Hammond, Ali Hindi, Kath Checkland

**Affiliations:** ^1^ Centre for Primary Care The University of Manchester Manchester UK

**Keywords:** continuity of care, healthcare professionals, patients, primary care networks

## Abstract

**Introduction:**

In England, primary care networks (PCNs) offer opportunities to improve access to and sustainability of general practice through collaboration between groups of practices to provide care with a broader range of practitioner roles. However, there are concerns that these changes may undermine continuity of care. Our study investigates what the organisational shift to PCNs means for continuity of care.

**Methods:**

The paper uses thematic analysis of qualitative data from interviews with general practitioners and other healthcare professionals (HCPs, *n* = 33) in 19 practices in five PCNs, and their patients (*n* = 35). Three patient cohorts within each participating practice were recruited, based on anticipated higher or lower needs for continuity of care: patients over 65 years with polypharmacy, patients with anxiety or depression and ‘working age’ adults aged between 18 and 45 years.

**Findings:**

Patients and clinicians perceived changes to continuity in PCNs in our study. Larger‐scale care provision in PCNs required better care coordination and information‐sharing processes, aimed at improving care for ‘vulnerable’ patients in target groups. However, new working arrangements and ways of delivering care in PCNs undermine HCPs' ability to maintain continuity through ongoing relationships with patients. Patients experience this in terms of reduced availability of their preferred clinician, inefficiencies in care and unfamiliarity of new staff, roles and processes.

**Conclusions:**

New practitioners need to be effectively integrated to support effective team‐based care. However, for patients, especially those not deemed ‘vulnerable’, this may not be sufficient to counter the loss of relationship with their practice. Therefore, caution is required in relation to designating patients as in need of, or not in need of continuity. Rather, continuity for all patients could be maintained through a dynamic understanding of the need for it as fluctuating and situational and by supporting clinicians to provide follow‐up care.

**Patient and Public Involvement (PPI):**

A PPI group was recruited and consulted during the study for feedback on the study design, recruitment materials and interpretation of findings.

## 1 INTRODUCTION

Primary care provision has been undergoing reorganisation in several countries over the past two decades.^1^, ^2^ In the United Kingdom, the policy response to increasing patient demand and general practitioner (GP) workforce pressures putting strain on general practice has been to foster collaboration between practices in the form of primary care networks (PCNs).^3^ Internationally, collaborative network models for primary care aim to improve care coordination for target populations, increase emphasis on preventative and population health, improve management of chronic conditions and reduce hospital admissions,^2^ improve outcomes, sustainability, quality of care, efficiency and health equity.^1^ The implementation of PCNs in English general practice involves larger‐scale healthcare provision and the introduction of a wider range of healthcare professional (HCP) roles, in part through the Additional Roles Reimbursement Scheme (ARRS) (https://www.england.nhs.uk/gp/expanding-our-workforce/).

Continuity of care has been the bedrock of general practice in the United Kingdom since its inception,^3^ but it has been eroding.^4^ There are concerns that care provision on a larger scale and involving a wider range of HCPs may be more fragmented and could undermine continuity of care.^4^, ^5^ This is because while protocols guide treatment, management continuity, which involves care coordination and relies on information sharing, implies knowing how, when and with whom to get advice, ask others to take action in a patient's care, get feedback on a patient's treatment from others and share important information about a patient.^6^ Much of this work within practices may be informal, however, HCPs and practice staff may no longer be colocated and protocols cannot cover all scenarios, therefore coordination of care is an important aspect of continuity to attend to in the context of PCNs.

### 1.1 The introduction of PCNs in England

The reorganisation of general (family) practice in England around PCNs, introduced in 2019^7^ entails new modes of collective delivery of primary care services through networks of practices, to exploit their scale and opportunities for collaboration.^3^, ^8^ The expanded skill‐mix provided by ARRS includes roles, such as physician associates, social prescribers and clinical pharmacists (https://www.england.nhs.uk/gp/expanding-our-workforce/). These roles could extend the range of services ‘upstream’ in primary care and make existing services more sustainable,^7^ maintaining adequate provision of appointments.^9^

The focus of these changes has, to date, emphasised patients' access to care,^3^, ^4^ framed in relation to providing timely appointments, for example, by diverting patients with minor illnesses to clinicians other than GPs.^7^ New PCN‐level modes of care provision include extended access hubs and clinics for chronic conditions that can address more of patients' needs in a single visit.

### 1.2 Continuity of care in general practice

Continuity of care has been a defining feature of primary care services, particularly within UK general practice.^4^ Continuity of care matters, not only for patient satisfaction,^10^ but also for a range of health outcomes,^11^ including patient mortality, and benefits to the health system through reducing unplanned hospital presentations.^4^

Continuity has been conceptualised in terms of multiple intersecting dimensions, with Haggerty's^12^ widely used model proposing three dimensions: relational, management and informational continuity. Relational continuity refers to the ongoing relationship between an individual clinician and patient; management continuity to ‘behind the scenes’ work to coordinate care that extends beyond a single visit, care setting or clinician; and informational continuity to sharing relevant history about the patient to ensure clinicians have the necessary information to treat them safely and effectively.

There is evidence to suggest that continuity is particularly important for certain groups of patients and conditions, including chronic conditions^13^ and vulnerable patients with multimorbidity.^14^ Relational continuity, in particular, facilitates management and informational continuity^13^ and generates patient trust and confidence in care received,^10^, ^11^ which can facilitate disclosure of health issues by the patient.^4^

The research evidence reflects different dimensions of continuity. Management and informational continuity predominate for chronic care that cuts across care settings,^13^ whereas relational continuity is often more highly valued by patients with mental health issues.^15^ In practice, however, as in the research literature, continuity of care is commonly framed, implicitly or explicitly, in terms of *relational* continuity.^16^ It emphasises patients being able to see the same GP or HCP for follow‐up appointments within an episode of care.^15^ While continuity in primary care has been framed in relation to services provided by GPs and nurses in ‘traditional’ general practices, there has been little attention paid in the research literature to how the delivery of primary care through PCNs, at scale and with expanded skill mix, influences continuity of care. Nor has consideration been given to whether different aspects of continuity may be enhanced or undermined by these changes. To address these questions, our paper explores how the dimensions of continuity of care are influenced by the development of PCNs in England.

## 2 MATERIALS AND METHODS

Data reported in this paper are drawn from a mixed methods study comprising a patient survey and interviews with HCPs and patients of participating PCNs. The study sought to investigate patients' and HCP' views and experiences of continuity of care and how they have been affected by organisational and workforce changes in the development of PCNs.

The study received ethical approval from the University of Manchester REC and the HRA London SouthEast REC (21/PR/0079).

### 2.1 Sampling

Five PCNs (sites A–E) were recruited by purposive sampling for maximum diversity in geographical location, rurality, deprivation and size (see Table 1). Practices within those PCNs were subject to convenience sampling through invitation by PCN research leads.

**Table 1:** Characteristics of participating primary care networks.

PCN	Region of England	Location	No. of practices	Participant practices	Practice list sizes (approximatley)	Deprivation decile (1 = more deprived, 10 = less deprived)
A	London	Large city with mainly affluent practice populations.	6	A1	∼9500	4/10
				A2	∼1300	9/10
				A3	∼7500	8/10
				A4	∼8000	8/10
B	North West	Large city with deprived to very deprived practice populations.	18	B1	∼8500	5/10
				B2	∼7000	6/10
				B3	∼7000	7/10
				B5	∼7000	8/10
C	North East and Yorkshire	Mixed urban and rural with very affluent populations in participating practices.	12	C1	∼14,500	10/10
				C3	∼5500	10/10
				C4	∼3500	10/10
D	North East and Yorkshire	Mainly rural with both very deprived and very affluent practice populations.	8	D1	∼14,000	2/10
				D2	∼12,000	2/10
				D3	∼13,000	9/10
				D4	∼10,000	9/10
E	Midlands	Small city with mainly very deprived practice populations.	9	E1	∼2000	8/10
				E2	∼4500	6/10
				E3	∼7000	9/10
				E4	10,000	9/10

### 2.2 Patient and public involvement (PPI)

A PPI group was recruited from the University's PRIMER group and members of the public via advert with research for the future. PRIMER provides a resource to facilitate PPI input into research from which members were engaged.

The PPI group provided feedback on the patient questionnaire, patient interview topic guide, analytical themes and findings. As patients are unable to directly experience management and informational continuity, the PPI group supported greater attention to how this translated into patients' ‘felt experience’, for example, noting that past experiences of primary care influenced patients' expectations of continuity we created a coding theme for disconnects in their expectations.

### 2.3 Data collection

This paper uses qualitative data from interviews with HCPs (*n* = 33) across 19 practices in the five study PCNs, and patients of those practices (*n* = 35). Semi‐structured interviews were conducted by researchers M. G. and A. H. between December 2021 and June 2022 by telephone or video conferencing. GPs and other HCPs from participating practices were invited to participate in a telephone interview through practice gatekeepers (see Table 2).

**Table 2:** Healthcare professional participant roles and numbers.

Role	Description	Numbers
GP	General practitioner, family physician (used interchangeably).	15
Nurses (including nurse clinicians, senior practice nurses and advanced nurse practitioners)	Advanced nurse practitioners are healthcare professionals who have developed higher‐level skills with expanded roles and scopes of practice.	6
Clinical pharmacists	Support GPs and other HCPs and may work with patients to advise on managing their medicines.	5
Pharmacy technician	Works under the supervision of a pharmacist.	1
Physician associates	Have generalist medical training, providing care under the supervision of a doctor.	2
Healthy ageing coordinator	Proactively identifies and works with vulnerable older patients to identify needs for support and coordinates care across health and care services.	1
Patient ambassador	Supports patients 1*–*1 to identify care and support needs and facilitates care navigation (similar to social prescribers).	1
Network mental health lead	Mental health practitioners who address biopsychosocial needs of patients with mental health problems, as part of a multidisciplinary team.	1

Abbreviations: GP, general practitioner; HCP,  healthcare professional.

Patients with polypharmacy,^17^ mental health issues^15^ and those aged 65 and older^18^ benefit more from relational continuity. Conversely, quicker access to care is traded‐off against continuity of care most by younger patients; men aged 17–45 and women aged 17–35.^19^ Therefore, we recruited patients from participating practices from three cohorts anticipated to have higher (1 and 2, below) or lower (3) needs for continuity of care: (1) patients over 65 years with polypharmacy, (2) patients with mild to moderate anxiety or depression and (3) adults aged 18–45.

A sample of each of the three cohorts of patients from each practice (*n* = 30; total sample size = 362) identified through a search of patient records by practice staff was sent a questionnaire. The questionnaire pack included an invitation to further participate in an interview. Patient interviews by cohort are shown in Table 3.

**Table 3:** Patient interview participants by cohort.

Patient cohort	Numbers	Code
Ages 18–45 years (‘working age’)	8	W
Aged >65 with polypharmacy	19	P
Mild–moderate anxiety/depression	8	X

Interviews sought experiences of continuity of care, elements considered important, changes in the context of PCNs and, for patients, their wider experiences with practices. Interviews with HCPs additionally sought experiences of new working arrangements for providing care, with a wider range of HCPs, and how continuity is being supported.

Interviews of 20–60 min were audio‐recorded with participants' permission. Recordings were transcribed verbatim, anonymised and uploaded into NVivo v12 to support analysis.

### 2.4 Data analysis

Data were deductively and inductively analysed using thematic analysis^20^ to identify patterns. Initially, a set of codes reflecting themes in the research questions were established: dimensions of continuity provided or experienced, using Haggerty.^12^ (1) conceptualisations of relational, management and informational continuity, (2) perceived changes to continuity, (3) understandings of continuity, (4) mechanisms used to maintain continuity of care, (5) experiences of failures or threats to continuity, (6) when continuity is more important and (7) relationships between access and continuity of care. Further codes were developed by M. G. and A. H., accommodating subcodes of the original codebook and codes generated inductively for themes emerging from the data.

M. G. and A. H. coded a sample of five transcripts each, codebooks were compared, discussed with the wider team and refined by combining similar codes under relevant themes. A. H. brought patient‐facing and M. G. social science and primary care research expertise that supported themes to emerge actively through discussion and reflection with the wider study team, knowledge of the literature and input from the PPI and advisory groups. The inclusion of additional codes was based on their value in addressing the research questions, with the PI, S.J. making the decision in the case of uncertainty or disagreement. Coding was continued by M. G. alone and discussed with the study team, PPI and advisory groups.

Quotes are attributed to patients by PCN (A–D), practice number (e.g., B1 is practice 1 in PCN B) and patient cohort (W, X or P, see Table 3) and patient number (e.g., B1X52). Quotes from HCPs are attributed by PCN code, practice number, role and two‐letter pseudonym.

## 3 RESULTS

Continuity of care, understood largely as relational continuity, was regarded as important to most HCPs and patients as ‘seeing the same HCP’ (usually a GP). HCPs acknowledged that it is now harder for patients to get continuity of care, that they continue to provide continuity for vulnerable patients, and that all patients can request to see a preferred HCP.

Many patients, regardless of cohort, reported finding it harder to get an appointment with their usual or preferred GP than in the past, translating into poorer relational continuity, while some were happy to see any GP. For patients who did not prioritise continuity, trust in the GP's expertise and awareness that they could access electronic patient records generated confidence in the care they received. Similarly, some HCPs were less concerned about diminishing relational continuity, believing informational and management continuity sufficient to provide good care and relational continuity unnecessary for most patients.

Informational continuity was, to a certain extent, considered by HCPs to be assured by the use of electronic patient record systems that enable patients' records to be accessed across the practice, important information to be ‘flagged’ and task requests to be exchanged by HCPs involved in the patient's care, supporting care coordination.

The findings are structured according to three key themes arising from the data: ‘providing primary care at scale’, ‘integrating new types of HCP’ and ‘mechanisms for providing continuity of care’.

### 3.1 Providing primary care at scale

New ways of providing care were reported, using the clinical workforce to conduct consultations across multiple practices in the PCN rather than solely their own practices. For example, using pooled GP resource to provide remote (telephone) appointments for patients from any of their practices. This supported the provision of appointments but also undermined relational continuity by making it less likely that patients could book an appointment with their preferred GP.

I know they are very busy, but they also work on multiple sites, so it is not easy necessarily to track where they are, when they are going to be around. (A1P67)

For that 15 minutes I am dealing with different practice patients, not my patients. For the outcome of this I am, again, sacrificing the continuity of care of my patients. (E4‐GP‐BD)

In this way, HCPs, particularly GPs, expressed commitment to the idea of continuity even when challenging to provide. One patient articulated the loss of relational continuity in the context of the larger scale of the PCN:

It's got too big, they can't possibly know all the patients, and they're all moving from site to site. (D1P189)

For patients, losses of relational continuity were experienced as ‘not seeing the same GP twice’ and consequently having to repeat themselves:

It's very difficult to see the same person twice so that one tends to be repeating information. (B2P21)

To make care provision more sustainable, participating practices shared clinical workforces to provide sickness or leave cover for practices within the PCN. This also took clinicians away from their own practices and patients:

It's about cross‐cover and that's when the continuity will fall down if people are having to cover at other practices, then they're not going to be their own patients. (D1‐Advanced‐Nurse‐Practitioner‐AS)

Unlike GPs and established HCPs, participating ARRS practitioners had various working arrangements because they were a shared resource within PCNs. Some worked in all practices in their PCN, whereas others were assigned to a subset of practices. This meant that ARRS practitioners were available only part‐time at any given site and less able, in practice, to provide follow‐up consultations and to develop relationships with patients who were frequent attenders.

I just try to do as much, continuing to see them in my own practice, following them up as I can. (A2‐Physician‐Associate‐AC)

Patients in our study experienced fewer consultations with non‐GP practitioners, providing less evidence of relational continuity with them. However, those with chronic conditions who saw specialist nurses, and patients with young children experienced relational continuity with practice nurses through more regular contact.

I see the nurse, so she does a lot of the childhood things and I've seen the nurse for a lot, [nurse's name], I've seen her a lot for [child's name], if I need to check his ears or something, and she is amazing. (B2W4)

This meant that practitioners' roles influenced their capacity to provide relational continuity.

### 3.2 Integration of new types of HCP

Certain roles in participating PCNs, including the patient ambassador, network mental health coordinator and healthy ageing coordinator, involved one‐to‐one case work or group work with patients over periods of weeks or months. This meant they could provide time‐limited relational continuity, tending to preclude longer‐term continuous ‘cradle to grave’ relationships.

We designed just an 8 week program. However, patients were engaging so well with that, coming back week after week. (C‐Network‐Mental‐Health‐Practitioner‐AN)

HCPs reported that the expanded workforce, and staff turnover, created challenges for embedding new staff due to some roles having less time at any given practice, slowing the process of relationship building with other staff. Some staff did not know who to ask for help, how to work with specific individuals or lacked understanding of different practices' processes, including ARRS staff establishing how to work with GPs who referred patients to them, and validate their care decisions.

We get too big, and sometimes there's high staff turnover. And so where staff are leaving and joining they don't necessarily have the time to bed in and understand the processes fully. (A2‐Clinical‐Pharmacist‐AD)

Day‐to‐day working, being in this wider team, it's been a little bit unsettling to begin with, because all of a sudden we're working with very new GPs that we didn't work with before. So they didn't realise how I, personally, worked and where my background and skills base and competencies are. So that's taken a little bit of getting used to, because usually you can go in and work with somebody, and then they'd know what you're about, and what you can do, whereas I'm not always working with the same ones, so it's like, I've really got to say, this is what I do, this is what I can see, this is my background. So that's been a little bit disjointed, because obviously they've got to be able to see what I can do, and trust what I can do. (D3‐Advanced‐Nurse‐Practitioner‐AX)

In this respect, ARRS roles may experience a lack of relational continuity with other, particularly established HCPs in the PCN, in terms of familiarity generating mutual trust and understanding of how to work together. As such professional relationships and integration were perceived as important in informational and management continuity.

### 3.3 Mechanisms for providing continuity of care

HCPs acknowledged threats to and losses of relational continuity for patients and reported various mechanisms used in their PCNs to preserve continuity of care for patients for whom they believed continuity was important.

It's tremendously important but it's increasingly rare. We try and … because of the way we run our planned care clinics, we can book our own follow‐ups and provide continuity of care but it is really a minority of patients now who are getting continuity of care, those with complex needs, palliative patients and so on, rather than the majority. (D3‐GP‐AW)

HCPs in all PCNs reported formalisation of processes to preserve continuity for vulnerable patients, including the introduction of care coordinators, care coordination hubs or patient ambassadors, facilitated by vulnerable patient lists. Patients considered ‘vulnerable’ included those with learning difficulties or mental illness, frail elderly patients, palliative patients, those with multiple comorbidities, chronic conditions or serious illness. They also included patients who had broader, intersecting health and care needs, such as those with unstable or complicated living circumstances, or where there were safeguarding concerns.

We have a vulnerable patients list and if we think somebody's vulnerable in whatever, they could be young or old, vulnerable to anything one of us will put them on what we call the vulnerable patients list …. it could be they're in an abusive relationship, it could be their child is at risk, it could be it's an elderly person. (B2‐Nurse‐Clinician‐AK)

The Healthy Ageing Coordinator and Patient Ambassador supported these kinds of biopsychosocial needs and linked patients to other HCPs and wider services. One PCN had care coordinators working from a ‘hub’ supporting similar activity.

HCPs reported greater emphasis on robust documentation, access to electronic patient records and regular meetings for effective information sharing, and communication within the PCN to maintain continuity. All participating practices used electronic patient records, however, with several software systems used in England, they did not necessarily use the same system as others in their PCN, presenting interoperability challenges for PCN‐wide data sharing in one participating PCN and occasional failures in sharing patient information. Being able to view the electronic medical records of patients from other practices in the PCN when treating them was considered important to informational continuity.

In some PCNs, specific patient groups, including those in care homes, benefited from dedicated HCPs, which improved relational continuity. ‘One stop shops’ for certain conditions were developed in other PCNs, which could facilitate relational continuity with specialists such as diabetes nurses.

If somebody has a cardiovascular problem together with diabetes, then you can do everything in a one‐stop clinic and that improves the continuity of care for the patients. (E2‐GP‐BB)

However, some patients outside target groups experienced worse management and informational continuity:

A lot of the time … for example, for referrals, I have to chase up to find out if referrals have gone through. (A3W292)

As individuals HCPs reported mitigating reduced clinical time in their practices by informing certain patients of their availability or booking follow‐up appointments during consultations. These routines tacitly acknowledged the challenges patients can face in getting timely follow‐up appointments with the same GP.

I say, well, these are the days I work and if you're on the duty list, if you ask for me and I'm available, I'll call you. (D3‐GP‐AW)

## 4 DISCUSSION

We asked how the development of PCNs has affected provision and experiences of continuity of care. The introduction of PCNs had multiple objectives. Meeting objectives related to scale, resource sharing and greater collaboration in the form of sharing staff and implementing working arrangements across a wider footprint reduced opportunities to provide relational continuity.

For many patients, the broader workforce may support PCN objectives related to improving access to appointments and moving services into the community while also reducing relational continuity. The exception is patients with chronic conditions who could develop relational continuity with specialist HCPs such as diabetes nurses. In this respect, PCNs may be meeting their objectives to support better care coordination and services integration for chronic conditions. The PCN objective of greater collaboration between practices and services integration necessitates increased information sharing and coordination of care, which support informational and management continuity. This was apparent in our study PCNs in more formalised and structured information sharing between practices. From the perspective of objectives of PCNs' development internationally, better‐coordinated care, particularly for chronic conditions provided more formalised care for patients with target conditions, thereby improving management continuity.

### 4.1 Implications for patients

Our findings indicate that continuity of care in the form of relational continuity is no longer experienced by most patients, echoing the widespread contention that it may be disappearing in the current UK context.^4^ Rather, HCPs directed efforts at maintaining continuity for certain groups of patients and types of conditions, implying categorisation of patients by the extent to which their effective diagnosis or treatment depends on continuity.^5^

While many patients noted a decline in continuity, some patients from the older cohort who were frequent users of primary care were able to see a preferred GP easily and remained satisfied with continuity of care. As more frequent users of primary care, they would have more opportunities to develop relational continuity with one or more HCPs.^13^

Losses of relational continuity may result in failure to recognise less overt or fluctuating needs for continuity or lead to inefficiencies when patients do not need continuity. In this study, some patients who believed they needed relational continuity did not receive it. Conversely, not all patients falling into the ‘vulnerable’ category needed or wanted relational continuity. This highlights that patients' expectations of continuity map onto their perceptions of the seriousness of their health issue at the time, which is dynamic rather than tied to specific patient groups.^5^

Losing relational continuity may make patients less inclined to disclose health issues during a consultation, less likely to discuss symptoms^11^ or may discourage them from seeking care.^16^ For HCPs, loss of relational continuity may reduce the quality of information available to them when treating a patient and could entail poorer care.^13^, ^16^ Loss of relational continuity may have little impact on patients who need only occasional, acute care from their general practice or patients who have no desire or need for relational continuity, such as those Kuipers et al.^14^ call the ‘prepared proactive patients’.

The more diverse workforce in participating PCNs expanded ‘in‐house’ services that could enhance the quality of care for some patients. However, this workforce may not enhance relational continuity because as specialists (e.g., clinical pharmacists) or generalists with a limited scope of practice (e.g., physician associates), they consult with a narrower set of patients.

However, loss of relational continuity is not inevitable if practices offer patients a choice of HCP when they book an appointment,^9^ and facilitate follow‐up appointments within clinical schedules.^15^

### 4.2 Implications for practices and PCNs

The team‐based care model that is emerging in PCNs generates a requirement for ongoing integration of ARRS staff at practice and PCN levels to build effective working relationships that can support the collaborative care objectives of PCNs.

Collaborative and larger scale, multidisciplinary care provision demands formalisation of processes that may previously have operated informally within a practice operating as an independent provider. In so doing they replicate the challenges of maintaining continuity across the wider health system, such as between general practice and acute services.^6^ While some PCNs characterise themselves as being like ‘one big practice’, other PCNs in our study may have integrated constituent practices far less and may continue to operate more independently. The latter may face challenges in providing management and informational continuity across practices' organisational boundaries. Even for well‐integrated PCNs, scale matters for continuity of care,^4^ and larger PCNs with more practices may have to do more work to provide continuity of care. Highly integrated PCNs may gain organisational benefits from larger‐scale care delivery, addressing efficiency and sustainability objectives and providing better‐coordinated care to vulnerable patients.^6^ However, they may create more onerous processes of getting care for other patients and undermine the ability to provide relational continuity to patients in any given practice.

These issues will be even more important to consider should the suggestions for the further integration of primary care set out in the Fuller Stocktake report^8^—including the emphasis on provision through neighbourhood‐based integrated teams—be pursued.

Most HCPs equated informational continuity with the effective use of electronic patient records. Nonetheless, formal information sharing is accompanied and underpinned by informal mechanisms facilitated by relationships between staff. There are enduring differences of opinion about whether informational and management continuity alone could provide sufficient continuity of care. Our study suggests that they cannot substitute for patients' ‘felt experience’ of continuity and also depends on patients' needs and expectations of care,^10^ and the extent to which the relationship with an HCP is therapeutic,^16^ and therefore the patient's health issues and personal circumstances.

#### 4.2.1 Limitations

The study uses solely qualitative interview data, which provides a detailed snapshot of perspectives on changes in how continuity of care is provided by HCPs and experienced by patients. The findings are limited to the five PCNs and 19 practices that participated in our study. Nonetheless, by exploring in‐depth the experiences of diverse PCNs and their patients, they highlight challenges in the provision of continuity of care applicable more broadly to PCNs in England.

Patient interviewees were not recruited evenly across PCNs and practices, meaning they are not representative of all patients' experiences in participating PCNs. There was no relationship between the level of deprivation and the number of patient interviewees recruited (see Supporting Information S1: Appendix 1).

#### 4.2.2 Contributions

Addressing a gap in knowledge about the impact of PCNs on continuity of care, our paper contributes evidence about implications for networks, practices and patients, and recommendations relevant to policy and practice.

By understanding continuity from diverse stakeholder perspectives, the study demonstrates that different aspects of continuity come to the fore at different times and circumstances within experiences of getting or giving care. Further research is required to understand how relational continuity can be supported through team‐based care at the PCN scale.

## 5 CONCLUSIONS

The capacity to provide relational continuity at a larger scale and with expanded workforces in PCNs depends on professionals' clinical roles and how they are deployed. Because individual clinicians may or may not be able to provide relational continuity, team‐based approaches to care, in which continuity of different types, may be provided collectively may be a mechanism to sustain it.

Supporting effective integration of staff at practice and PCN levels is necessary to build working relationships that can support these management and informational continuity, particularly for team‐based care for vulnerable patients. However, this may fail to compensate for losses of relational continuity, which may be inevitable in larger primary care organisations in the absence of interventions that seek to specifically enhance it. Therefore, caution is required in relation to designating patients as in need of, or not in need of continuity. Rather, continuity for all patients could be maintained through a dynamic understanding of the need for it as fluctuating and situational and by supporting clinicians to provide follow‐up care.

## AUTHOR CONTRIBUTIONS

**Mhorag Goff**: Conceptualisation; investigation; formal analysis; project administration; writing—review and editing; data curation. **Sally Jacobs**: Conceptualisation; investigation; funding acquisition; writing—original draft; writing—review and editing; methodology; project administration; supervision; resources. **Jonathan Hammond**: Writing—review and editing; writing—original draft; validation. **Ali Hindi**: Investigation; formal analysis. **Kath Checkland**: Writing—review and editing; writing—original draft; validation.

## CONFLICT OF INTEREST STATEMENT

The authors declare no conflict of interest.

## Supporting information

Supporting information.

## Data Availability

The data that support the findings of this study are available on request from the corresponding author. The data are not publicly available due to privacy or ethical restrictions.

## References

[1] Agyemang‐Benneh A , Francetic I , Hammond J , Checkland K . Evaluating primary care networks in low‐income and lower middle‐income countries: a scoping review. BMJ Glob Health. 2023;8(8):e012505. 10.1136/bmjgh-2023-012505 PMC10432626 37580101

[2] Leslie M , Khayatzadeh‐Mahani A , Birdsell J , et al. An implementation history of primary health care transformation: Alberta's primary care networks and the people, time and culture of change. BMC Fam Pract. 2020;21(1):258. 33278880 10.1186/s12875-020-01330-7 PMC7718828

[3] Checkland K , Hammond J , Warwick‐Giles L , Bailey S . Exploring the multiple policy objectives for primary care networks: a qualitative interview study with national policy stakeholders. BMJ Open. 2020;10(7):e038398. 10.1136/bmjopen-2020-038398 PMC7337885 32624477

[4] Pereira Gray D , Sidaway‐Lee K , White E , Thorne A , Evans P . Improving continuity: the clinical challenge. InnovAiT. 2016;9(10):635‐645.

[5] Guthrie B , Wyke S . Personal continuity and access in UK general practice: a qualitative study of general practitioners' and patients' perceptions of when and how they matter. BMC Fam Pract. 2006;7(1):11. 16504130 10.1186/1471-2296-7-11 PMC1413534

[6] Breton M , Haggerty J , Roberge D , Freeman GK . Management continuity in local health networks. Int J Integr Care. 2012;12:e14. 22977427 10.5334/ijic.682 PMC3429137

[7] NHS England . General practice forward view. 2016. Accessed December 15, 2023. https://www.england.nhs.uk/wp-content/uploads/2016/04/gpfv.pdf

[8] Fuller C . Next steps for integrating primary care: Fuller stocktake report. 2022. Accessed October 4, 2023. https://www.england.nhs.uk/publication/next-steps-for-integrating-primary-care-fuller-stocktake-report/

[9] Department of Health and Social Care . Our plan for patients. 2022. Accessed November 21, 2023. https://www.gov.uk/government/publications/our-plan-for-patients/our-plan-for-patients

[10] Lautamatti E , Sumanen M , Raivio R , Mattila KJ . Continuity of care is associated with satisfaction with local health care services. BMC Fam Pract. 2020;21(1):181. 32887566 10.1186/s12875-020-01251-5 PMC7487808

[11] Sidaway‐Lee K , Pereira Gray OBE D , Harding A , Evans P . What mechanisms could link GP relational continuity to patient outcomes? Br J Gen Pract. 2021;71(707):278‐281. 34045259 10.3399/bjgp21X716093 PMC8163455

[12] Haggerty JL . Continuity of care: a multidisciplinary review. BMJ. 2003;327(7425):1219‐1221. 14630762 10.1136/bmj.327.7425.1219 PMC274066

[13] Gulliford M , Cowie L , Morgan M . Relational and management continuity survey in patients with multiple long‐term conditions. J Health Serv Res Policy. 2011;16(2):67‐74. 20592048 10.1258/jhsrp.2010.010015

[14] Kuipers SJ , Nieboer AP , Cramm JM . Views of patients with multi‐morbidity on what is important for patient‐centered care in the primary care setting. BMC Fam Pract. 2020;21(1):71. 32336277 10.1186/s12875-020-01144-7 PMC7184691

[15] Biringer E , Hartveit M , Sundfør B , Ruud T , Borg M . Continuity of care as experienced by mental health service users—a qualitative study. BMC Health Serv Res. 2017;17(1):763. 29162112 10.1186/s12913-017-2719-9 PMC5698968

[16] Reid J , Cormack D , Crowe M . The significance of relational continuity of care for Māori patient engagement with predominantly non‐Māori doctors: findings from a qualitative study. Aust N Z J Public Health. 2016;40(2):120‐125. 26337777 10.1111/1753-6405.12447

[17] Tarrant C , Lewis R , Armstrong N . Polypharmacy and continuity of care: medicines optimisation in the era of multidisciplinary teams. BMJ Qual Saf. 2023;32(3):121‐124. 10.1136/bmjqs-2022-015082 36216498

[18] Nutting PA . Continuity of primary care: to whom does it matter and when? Ann Fam Med. 2003;1(3):149‐155. 15043376 10.1370/afm.63 PMC1466596

[19] Salisbury C , Manku‐Cott T , Moore L , Chalder M , Sharp D . Questionnaire survey of users of NHS walk‐in centres: observational study. Br J Gen Pract. 2002;52(480):554‐560. 12120727 PMC1314357

[20] Braun V , Clarke V . Conceptual and design thinking for thematic analysis. Qual Psychol. 2022;9(1):3‐26.

