# Supplementary material for: Investigating the impact of primary care networks on continuity of care in English general practice: Analysis of interviews with patients and clinicians from a mixed methods study
Source: Health Expect. 2024 Mar 31;27(2):e14032. doi: 10.1111/hex.14032 (PMC10982586; doi:10.1111/hex.14032)
Supplement: Supplementary file 1 — Supporting information. [file HEX-27-e14032-s001.docx]

## **Appendix 1**

### **Table 4. Patient interview participants by deprivation level**

| **Participant practices** | **Deprivation decile**  **(1= more deprived, 10=less deprived)** | **Patient interviews** |
| --- | --- | --- |
| A1 | 4/10 | 3 |
| A2 | 9/10 | 2 |
| A3 | 8/10 | 1 |
| A4 | 8/10 | 2 |
| B1 | 5/10 | 1 |
| B2 | 6/10 | 5 |
| B3 | 7/10 | 0 |
| B5 | 8/10 | 2 |
| C1 | 10/10 | 0 |
| C3 | 10/10 | 2 |
| C4 | 10/10 | 0 |
| D1 | 2/10 | 5 |
| D2 | 2/10 | 1 |
| D3 | 9/10 | 2 |
| D4 | 9/10 | 0 |
| E1 | 8/10 | 0 |
| E2 | 6/10 | 3 |
| E3 | 9/10 | 5 |
| E4 | 9/10 | 1 |
